# Terrestrial Spatial Distribution and Summer Abundance of Antarctic Fur Seals ( *Arctocephalus gazella* ) Near Palmer Station, Antarctica, From Drone Surveys

**DOI:** 10.1002/ece3.70833

**Published:** 2025-04-03

**Authors:** Gregory D. Larsen, Megan A. Cimino, Julian Dale, Ari S. Friedlaender, Marissa A. Goerke, David W. Johnston

**Affiliations:** ^1^ Nicholas School of the Environment Duke University Beaufort North Carolina USA; ^2^ Ocean Sciences Department UC Santa Cruz Santa Cruz California USA; ^3^ Antarctic Support Contract Centennial Colorado USA

**Keywords:** Antarctica, drones, habitat suitability modeling, pinnipeds, polar ecology, population estimation, remote sensing

## Abstract

The shifting climatic regime of maritime Antarctica is driving complex changes across trophic levels that are manifesting differentially across its resident species and regions. Land‐breeding pinnipeds have increased their seasonal attendance near Palmer Station since the earliest observations in the mid‐1900s, and Antarctic fur seals ( *Arctocephalus gazella* ) now represent a significant but unstudied predator population in the region during the austral summer. To characterize the timing of abundance and the fine‐scale distribution of this seasonal attendance, we carried out regular drone surveys of terrestrial habitats near Palmer Station in the austral summer of 2020. Using repeat animal counts and photogrammetric data products, we modeled fur seal abundance at survey sites over the period of observation, modeled habitat suitability based on fine‐scale topographic habitat characteristics, and estimated abundance across terrestrial habitats near Palmer Station as a function of these products. High habitat suitability was most associated with low‐slope and low‐elevation inshore terrain and with relatively dry, sun‐exposed, and wind‐sheltered locations, and estimated peak abundance occurred on March 11 (day 71) of 2020. Models estimated 2289–5544 (95% confidence interval) fur seals on land across all potential terrestrial habitats (41 discrete sites) near Palmer Station and Wylie Bay on the south coast of Anvers Island during peak abundance. This constitutes a first estimate of the aggregate timing, abundance, and distribution of Antarctic fur seals in the terrestrial habitats of this region—a critical first step in understanding the phenology and ecological role of this largely nonbreeding predator population. These findings additionally establish a baseline from which to estimate future changes in this seasonal population and its effects on sympatric terrestrial and marine biota, as the physical environment and food chain of the western Antarctic Peninsula transform under long‐term climatic changes.

## Graphical abstract

A summer population of nonbreeding Antarctic fur seals arrives each year at the west Antarctic Peninsula, but remains poorly characterized amid the rapid climatic and ecological changes that are restructuring the region's environment and food web. We carried out a campaign of drone surveys to map the occurrences of Antarctic fur seals across a variety of habitats near Palmer Station, Antarctica, during a 3‐month period of observation, and we modeled both abundance over time and distribution across sites using drone‐derived seal counts and locations alongside satellite‐derived topographic products. Our findings characterize the timing and spatial associations of Antarctic fur seals in this part of the Antarctic Peninsula, and by combining observed abundance trends with habitat suitability modeling, we calculated a regional estimate of all Antarctic fur seals on land during peak abundance in 2020.

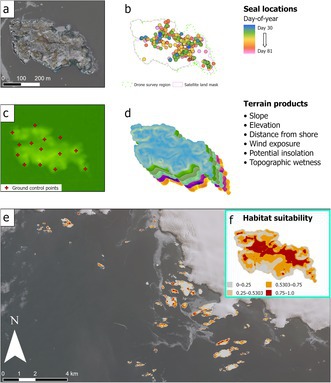

## 1 Introduction

The western Antarctic Peninsula (WAP) has experienced strong warming trends since the 1950s (Vaughan et al. 2003; Turner et al. 2005, 2016; Oliva et al. 2017; Carrasco, Bozkurt, and Cordero 2021), accompanied by responsive changes in Antarctic terrestrial biota (Smith 1994; Amesbury et al. 2017) and the marine community (Constable et al. 2014; Turner et al. 2014; Gutt et al. 2015). These changes are expected to affect trophic levels (Smith et al. 1999; Ducklow et al. 2013; Constable et al. 2014), and in recent decades, scientists have described large‐scale poleward range shifts in phytoplankton (Montes‐Hugo et al. 2009), zooplankton (Atkinson et al. 2004, 2019), and some megafauna (Forcada and Trathan 2009; Lynch et al. 2012). Such evidence emerges through multiple institutions and frameworks that monitor the Antarctic environment, including the Commission for the Conservation of Antarctic Marine Living Resources Ecosystem Monitoring Program, which monitors krill stocks by proxy through studies of krill predators (Agnew 1997), and the Palmer Long‐Term Ecological Research program, which studies long‐term ecological changes along the WAP (Smith et al. 1995, 2003).

**FIGURE A1:**
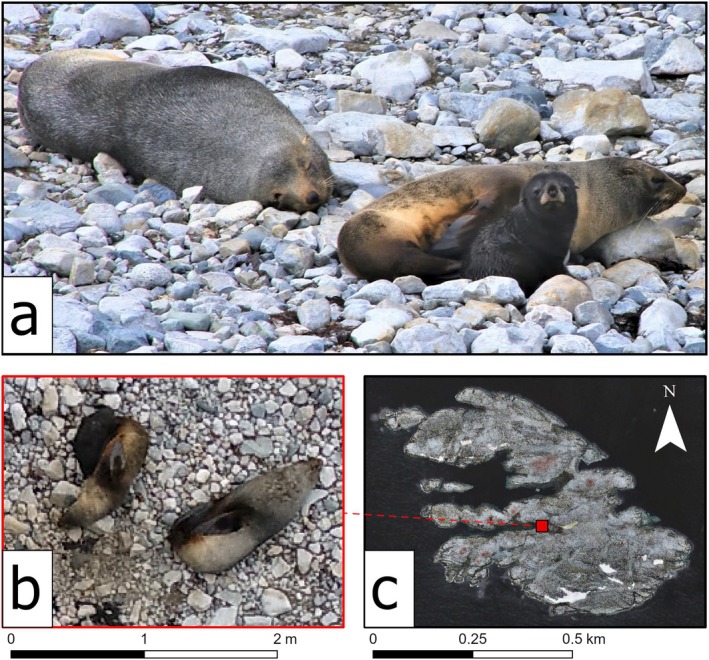
Photography and location of an Antarctic fur seal pupping near Palmer Station. Photography at ground level (a) and from a drone (b) documented a reproductive female associating with a pup near an attending male at a sheltered beach at Dream Island (c), on January 28, 2020. Ground imagery was collected using a Canon 6D camera with a 200 m lens (photo credit: Marissa Goerke), aerial imagery was collected using a DJI Phantom 4 Pro, and the reference basemap consists of a color orthomosaic collected on February 13, 2020.

Five pinniped species occur regularly at the south side on Anvers Island near Palmer Station (Cimino et al. 2023): crabeater seals ( *Lobodon carcinophaga* ), leopard seals ( *Hydrurga leptonyx* ), Weddell seals ( *Leptonychotes weddellii* ), southern elephant seals ( *Mirounga leonina* ), and Antarctic fur seals ( *Arctocephalus gazella* ). Of these, southern elephant seals and Antarctic fur seals constitute growing predation pressures in the marine ecosystem of the Palmer Archipelago, with both populations notably increasing since historical records. Southern elephant seals were noted in 1955 among the earliest recorded observations for the Palmer Station vicinity (Holdgate 1963), but local breeding was first recorded with two pups in 1982 (Heimark and Heimark 1986) and has grown to dozens of pups in recent years. Antarctic fur seals were first sighted in the area in the mid‐1970s, including four small breeding groups in the adjacent Gossler Islands (Parmelee et al. 1977), and thereafter expanded to a larger summer population during the molting season. This presence reached an apparent peak in 1994 (“Antarctic Specially Protected Area No 113 (Litchfield Island, Arthur Harbor, Anvers Island, Palmer Archipelago): Revised Management Plan” 2014), followed by a period of decline, according to opportunistic counting efforts (Siniff et al. 2008). Recently, a female and pup were observed closer to Palmer Station in 2020 (Figure A1), which is located far south of the larger colonies in the South Shetland Islands (~300 km away) and South Georgia Island (1900 km away), suggesting that breeding attempts may be expanding in this region—although sporadic attempts do not necessarily anticipate a successful rookery (Waluda, Gregory, and Dunn 2010). However, even in the absence of a breeding population, nonbreeding Antarctic fur seals still exert an ecologically significant pressure on the marine ecosystem, especially local krill stocks (Lowther et al. 2020), and on terrestrial communities, potentially trampling mosses (Lewis Smith 1988; Favero‐Longo et al. 2011) and disturbing ground‐nesting seabirds (Larsen et al. 2024), and the current status of this population is not well described.

There are many established methods to study the abundance and distribution of breeding Antarctic fur seals owing to predictable aspects of their reproductive biology: seasonally and spatially restricted availability of prey and habitat concentrate them at select rookery sites during discrete breeding seasons, high natal philopatry conserves rookery sites across generations, and breeding site fidelity allows the study of individual characteristics across years (Hoffman, Trathan, and Amos 2006; Hoffman and Forcada 2012). Regular foraging trips, characteristic of their income breeding strategy, also enable the deployment of biologging and telemetry devices with a high likelihood of recapture and tag recovery within seasons. Nonbreeding fur seals, by contrast, access comparatively diverse and diffuse terrestrial habitats for rest and molting during the latter summer months. Many nonbreeding haul‐out sites are seasonally occupied year‐after‐year (personal observation), but individuals have little known site fidelity within or across seasons, limiting the utility of identifying tags or archival data loggers that might never return to the site of deployment.

Unoccupied aircraft systems (hereafter, drones) can address some of these challenges of measuring aspects of a nonbreeding pinniped population (Larsen and Johnston 2024). Expansive spatial coverage can census individuals at low densities across complex topography and can furthermore test or validate the utility of monitoring index sites, such as key haul‐outs, for a nonbreeding population. Aerial censuses function similarly to counts from ground‐based observers in that they attempt to describe all animals occurring in the surveyed area at the time of observation (Hodgson et al. 2018)—highly mobile species present a high risk of repeat‐counting if they move between photographs (Brack, Kindel, and Oliveira 2018; Fust and Loos 2023), but this risk is low for hauled‐out pinnipeds. Aerial surveys can also introduce ambiguity or perception bias when observers visually interpret imagery from aerial perspectives (Brack, Kindel, and Oliveira 2018), potentially overlooking animals that are well camouflaged, especially in still photography. Ground‐based surveys, by contrast, can observe animals from various perspectives and observe movement over time, especially if animals are disturbed during the survey. However, this is a key advantage of aerial surveys over ground‐based methods: drone campaigns can observe wildlife and habitats while causing little or no anthropogenic disturbance to many animals (Borrelle and Fletcher 2017; Mulero‐Pázmány et al. 2017) or to nearby delicate, slow‐growing polar flora, such as Antarctic mosses and lichens (Tovar‐Sánchez et al. 2021; Raniga et al. 2024). Repeat surveys can be carried out with minimal infrastructure and limited human effort, and mapping from drone imagery can produce fine‐scale datasets of land cover and surface topography (Westoby et al. 2012), enabling spatial analyses that characterize species–habitat relationships. These technological advancements together enable high‐frequency monitoring and modeling of populations that were historically challenging or impossible to census (Krause and Hinke 2021). Such aerial methods are limited to operating under favorable weather conditions, requiring low wind and precipitation to a greater degree than ground‐based field methods, but they can also overcome common challenges of Antarctic research, like dense sea ice that commonly inhibits boat‐based transit and the deployment of ground‐based field teams.

In this study, we censused a population of predominantly nonbreeding or postbreeding Antarctic fur seals during the austral summer of 2020 with a drone survey campaign over selected coastal habitats of south Anvers Island. We produced a time series of counts and locations of terrestrial occupancy during these surveys, and we developed models to estimate the timing of seasonal abundance and habitat associations of the population, obtaining a local estimate of peak terrestrial abundance for this species. These findings represent a synthesis of methods enabled by high‐resolution remote sensing to characterize the summer phenology and distribution of this ecologically significant species of the Palmer Archipelago.

## 2 Material and Methods

### 2.1 Drone Surveys

**FIGURE 1:**
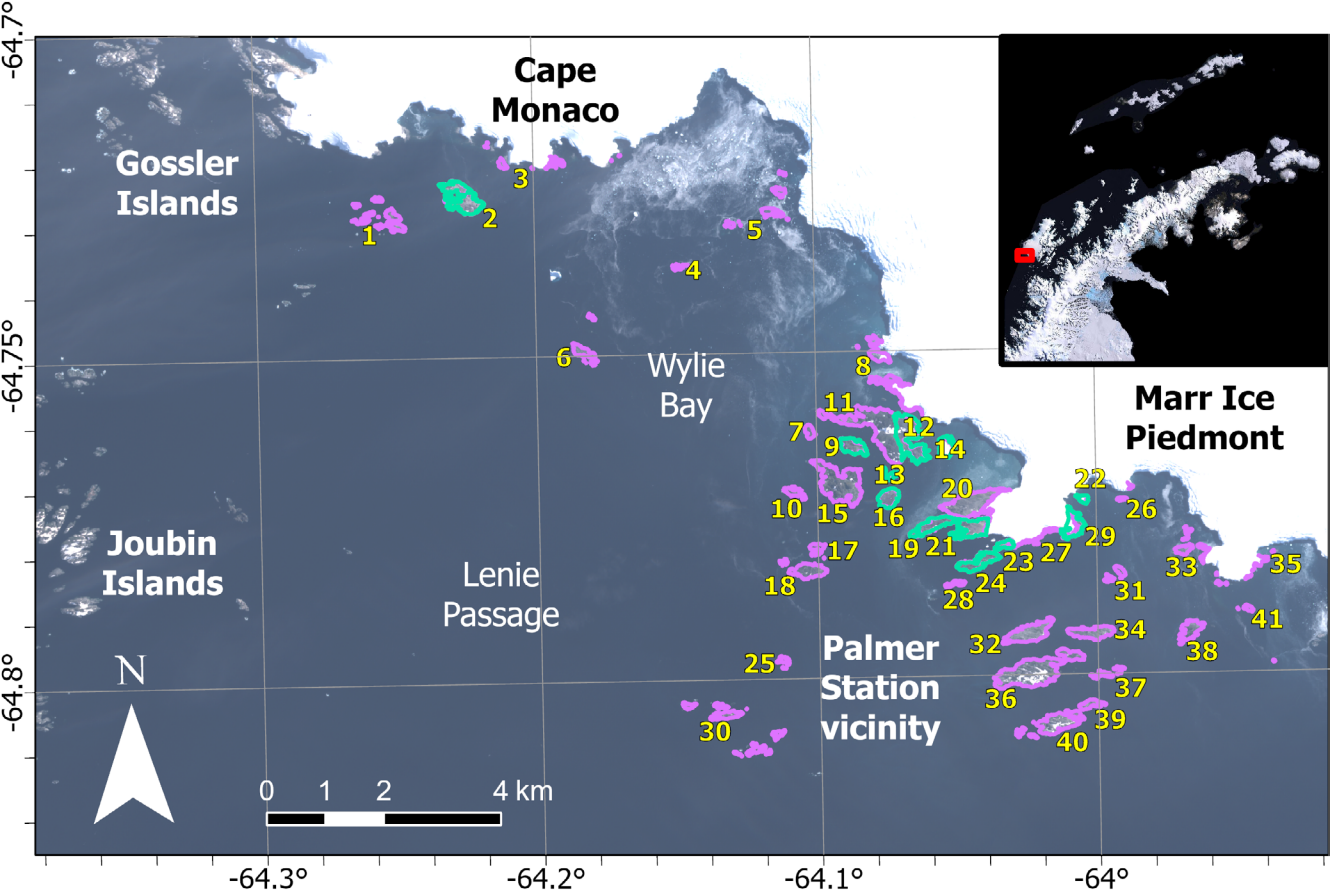
Coastal and offshore features and habitats of southwest Anvers Island. An inset (top right) shows the region's location and extent (red box) in the context of the WAP using the 2008 Landsat Image Mosaic of Antarctica (Bindschadler et al. 2008). Select locations were surveyed using drones (teal) for pinniped occupancy during summer 2020, and satellite‐derived elevation models were created for ice‐free coastal habitats (purple and teal) in the Palmer Station vicinity and Wylie Bay. Palmer Station is located at label “20” (Gamage Point), coordinates −64°46′, 64°03′. Numbered labels are listed in Table 1. Map projection: WGS 84/UTM zone 20S. Base imagery: Sentinel 2, true color, captured on February 18, 2020, with 25% transparency.

**FIGURE A2:**
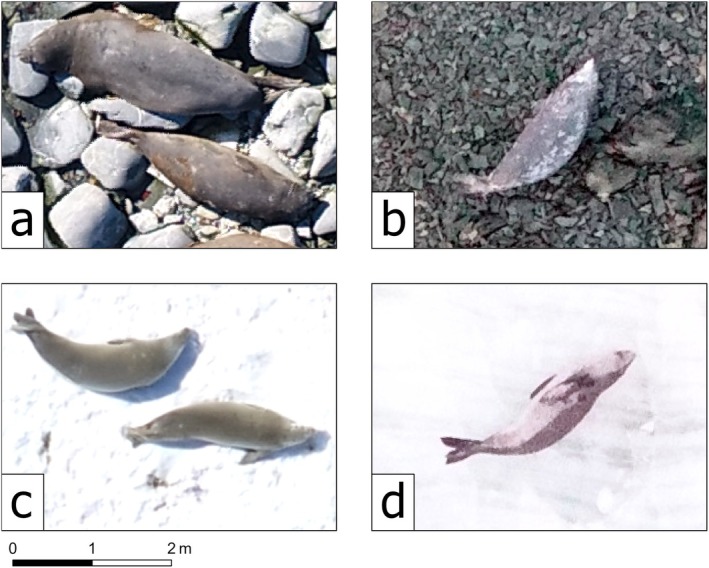
Example aerial photography of phocid species that commonly occur near Palmer Station. Examples are shown from surveys of land (a, b), snow (c), and adjacent sea ice (d) near Palmer Station and depict (a) southern elephant seals, (b) a Weddell seal, (c) crabeater seals, and (d) a leopard seal. Seals are shown at a consistent spatial scale and ground sample distances of 1.3–1.8 cm/pixel.

We carried out surveys of 14 sites near Palmer Station (Figure 1) between January 9 and March 23, 2020 using DJI Phantom 4 Pro quadcopter aircrafts with a default camera payload to collect uncalibrated color imagery (Appendix 1). Drones collected overlapping aerial photography along automated flight paths such that imagery could be processed into orthomosaic and photogrammetric products for counting and spatial analysis. Aerial photography was collected at target ground sample distances (GSDs) of 1.5–3 cm, such that individual seals could be discriminated against background substrates, classified to species by shape and color (Figure A2), located precisely within the landscape, and counted in aggregate. Survey sites were selected and prioritized based on their relative accessibility and past observations of pinniped occupancy from the authors and other personnel at Palmer Station. Survey times were selected opportunistically amid variable weather conditions and other concurrent research protocols but aimed for near‐weekly surveys of some sites near Palmer Station and ad hoc coverage of additional sites as possible. Drone operations caused no noticeable disturbance during flight at survey altitudes, but minor behavioral differences were observed during some drone launches and landings, and when accessing sites before drone operations. When these were observed, we selected new launch sites to minimize risk of further disturbance. All drone surveys were conducted under Antarctic Conservation Act permit ACA 2020‐016 and NOAA permit 14809‐03.

### 2.2 Satellite Imagery Selection and Evaluation

We obtained 2‐m resolution digital elevation models (DEMs) derived from imagery from WorldView‐series satellites in the DigitalGlobe (Westminster, CO) collection. DEMs were provided by the Polar Geospatial Center (University of Minnesota), and were generated using “surface extraction by TIN‐based search space minimization” (Noh and Howat 2017) from stereo‐paired scenes of satellite imagery. We visually inspected DEMs and selected two products that described our study regions without obvious errors, using imagery from 2012 (describing Dream Island, Casey Islands, and west‐facing exposed coastlines of north Wylie Bay) or 2019 (all other sites, Figure 1). We vertically corrected each DEM using ground control points (GCPs) that we collected across three sites (Appendix 2) and we removed all areas of water and glacier (Appendix 3).

### 2.3 Fur Seal Counts and Locations

We processed all drone surveys (*n* = 98) to orthomosaic products using Pix4Dmapper 4.3 photogrammetric software. For each site, we aligned orthomosaics to each another and our satellite base maps, such that all products shared the same geospatial extent and reference (Appendix 3). We then manually counted pinnipeds in each survey by systematically inspecting the orthomosaic and marking fur seal locations. Ambiguous detections were resolved by examining unprocessed aerial photos, and by toggling between orthomosaics from different dates to rule out persistent landscape features. Antarctic fur seals were easily discriminated from other pinniped species based on posture and morphology (Figures A1 and A2), and other pinnipeds were usually easy to discriminate from one another at 1.5 cm GSD. Discrimination between similar‐shaped phocid species was aided by pelage scars (crabeater seals), pelage patterns (Weddell seals), flipper morphology, and craniofacial morphology, as visible in drone imagery, but was generally less confident than discrimination between phocids and Antarctic fur seals.

### 2.4 Topographic Predictors

**FIGURE 2:**
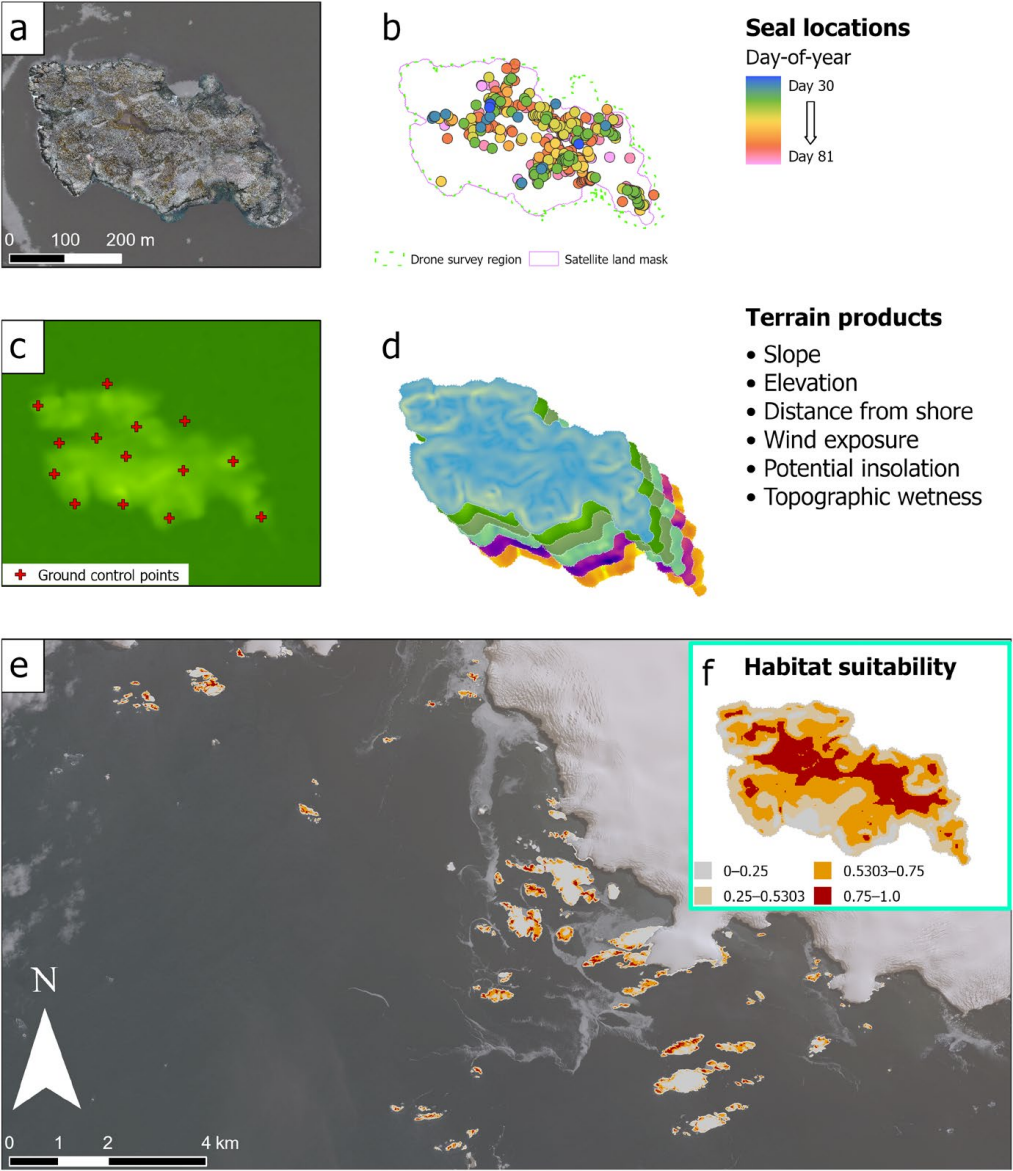
Spatial products from an example site (Humble Island). These include (a) an orthomosaic from a single drone survey, overlaid on WorldView‐2 satellite imagery, (b) identified locations of Antarctic fur seal occupancy throughout the 2020 season, (c) modeled elevation from WorldView‐3 satellite imagery, with GCP locations used to correct absolute error, (d) derived terrain products, (e) a prediction of the habitat suitability model across the entire study region, overlaid on WorldView‐2 imagery, and (f) an example subset of that prediction at Humble Island. Prediction values estimate habitat suitability from Antarctic fur seal locations in drone surveys conducted across 12 sites during days 43–45 and 68–73 in 2020, using a threshold (0.5303) that maximizes the sum of sensitivity and specificity for the habitat suitability model. Base imagery: WorldView‐2, true color, captured on February 28, 2019, with 25% transparency. DEM source: SETSM product from WorldView‐3, captured on November 19, 2019. Imagery 2019 Maxar.

**FIGURE A3:**
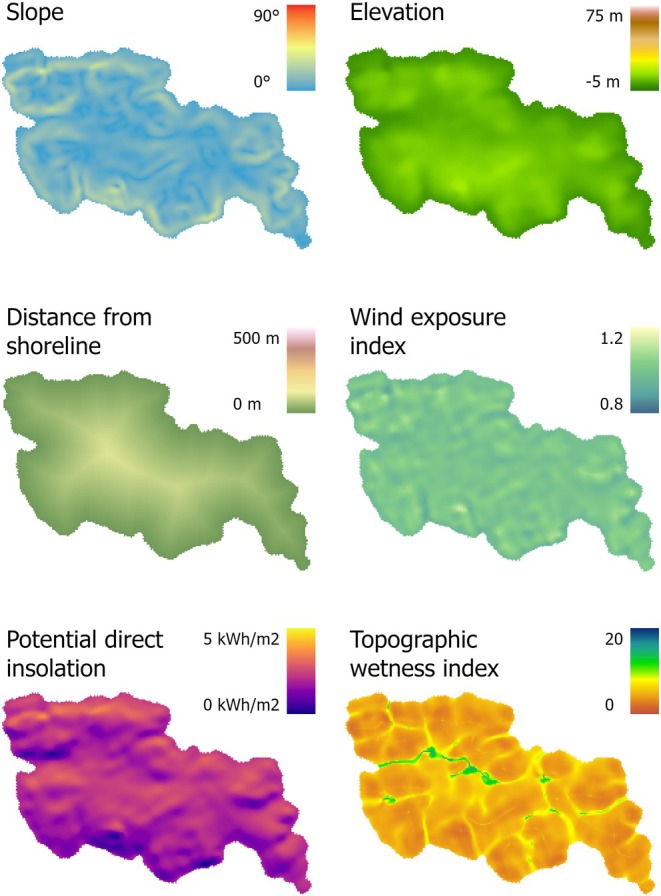
Terrain analysis products used for MaxEnt modeling, subset to the example site of Humble Island. Products are the same as shown in Figure 2d, but shown in full with legends.

We used satellite DEMs to generate maps of different topographic characteristics that could affect habitat suitability for Antarctic fur seals across the entire study region (Figure 2). We selected predictors that could challenge pinniped locomotion—elevation, slope, and surface distance from shoreline—and microclimate predictors that could mediate pinniped thermoregulation—potential direct insolation and wind exposure from all angles (examples in Figure A3). We included topographic wetness index (Beven and Kirkby 1979) as a possible predictor of both accessibility and microclimate. We did not include rugosity as a predictor, considering slope to be a mathematically simpler predictor that exerts similar functional limitations on pinniped locomotion and site occupancy. We also did not include marine predictors, such as local bathymetry, probable foraging regions, and potential access routes to haul‐outs. Such predictors would likely improve models of any terrestrial pinniped habitat, but they require additional data sources, models, and methods that were not available to us, are often unavailable entirely for these sites, and were ultimately deemed beyond the scope of this modeling effort.

We obtained elevation from the corrected and land‐masked DEMs, and we generated slope and surface distance using ArcGIS Pro 2.7.1. We generated other products using System for Automated Geoscientific Analyses (SAGA) 8.2.1 (Conrad et al. 2015) tools for “potential incoming solar radiation” (Böhner and Antonić 2009), “wind exposition index” (Böhner and Antonić 2009) and “topographic wetness index” (Böhner and Selige 2006). Distance from shoreline was significantly correlated with elevation (*r* = 0.79), but was not omitted because some types of species distribution models are not negatively affected by collinearity among predictors (Feng et al. 2019). We used surveys from 12 sites to model fur seal abundance and habitat suitability: Amsler Island East, Bonaparte Point, Dietrich Island, Dream Island, Elephant Rocks, Humble Island, Kristie Cove, Point 8 Island, Point 8, Shortcut Island, Shortcut Point, and Torgersen Island (Figure 1).

### 2.5 Spatial Habitat Suitability Modeling

We estimated approximate habitat suitability using maximum entropy (MaxEnt) modeling for species distributions (Phillips, Anderson, and Schapire 2006) with the “dismo” package (Hijmans et al. 2017) in R programming language version 4.0.2 (R Core Team 2020). MaxEnt is one of several methods that can be used to model species distributions using presence‐only data (Valavi et al. 2022), which is appropriate for vagile organisms, like pinnipeds, for whom a detected absence during photographic sampling reflects an absence at the time of sampling (pseudoabsence) and not necessarily a persistent spatial absence over time (Elith et al. 2011). MaxEnt achieves presence–background modeling by estimating the distribution of habitat covariates that fit species occurrence locations with otherwise minimal difference from a null model of randomly sampled background data, maximizing entropy within the constraints of occurrence data. A variety of possible response functions allows MaxEnt to model complex species–habitat relationships within statistical bounds that limit overfitting. Among many current presence‐only methods, MaxEnt achieves high performance across different modeling scenarios, although alternative methods can yield better models for individual scenarios (Valavi et al. 2022).

We evaluated MaxEnt models using the area under the receiver operating curve (AUC) measurement, where 1 represents a model with perfect discrimination between presences and absences and 0.5 indicates a model that performs as well as a random selection. AUC is a prevalent metric for evaluating MaxEnt models, independent of the threshold used to classify habitat suitability from model predictions (Mcpherson et al. 2004). We therefore fit a MaxEnt model, determined its AUC, and then evaluated its performance using a *k*‐fold cross‐validation resampling procedure, which randomly divides occurrence locations into *k* equally sized groups or folds of data, fits the model on *k* − 1 folds, and predicts the model onto the withheld fold. The predictions generated from the withheld fold are then used to evaluate the performance of the model (Elith et al. 2011). We used four replicate runs that partitioned 75% of the seal occurrences to fit models and 25% of the occurrences to validate the models. We also evaluated which environmental predictors contributed most to fitting the model by using a jackknife test for predictor importance, which, for each predictor, fits an alternative model with all but that withheld predictor and an alternative model with only that predictor, comparing the AUC among alternative models and the full model. Similar studies have used these modeling and evaluation methods to predict the distributions and habitat suitability of Antarctic predators at larger spatial scales on the WAP (Friedlaender et al. 2011; Cimino et al. 2013, 2016).

We selected two periods of 3–6 days around the dates of highest abundance during which we had surveyed the same 12 sites, allowing for a large number of seal presence locations while minimizing sampling bias and temporal autocorrelation in habitat suitability predictions (Table 1). Using 1726 animal locations from these sites and dates, we generated a model of habitat suitability using MaxEnt with all six topographic predictors hypothesized to influence fur seal spatial habitat selection (Figure 2). We then predicted this model across the Palmer Station vicinity (Figure 1) using the satellite‐derived DEMs to estimate topographic suitability across ice‐free coastal habitats. We classified habitat as “suitable” or “unsuitable” using the value that maximized the sum of sensitivity and specificity (maxSSS), 0.5303, as a classification threshold, based on the robust predictive performance of this objective threshold when applied to models with presence‐only data (Liu, White, and Newell 2013), and we evaluated this threshold using the true skill statistic (TSS) of the resulting classification (Allouche, Tsoar, and Kadmon 2006), where 1 represents perfect classification and a value ≤ 0 is no better than random. We then calculated the area of suitable habitat for each site in our study region using this threshold classification.

**TABLE 1:** Sites and metadata of all drone surveys (*n* = 98) for pinniped presence in the south Anvers Island region in 2020.

Site name	Coordinates	Area (km^2^)	Survey dates (day‐of‐year)	Pinniped species identified
Amsler Island East^12^	S 64.738°, W 64.065°	0.286	19, 32, 40, 45, 51, 56, 62, 69, 74, 80	*M. leonina* , *A. gazella* , *L. carcinophaga* , *L. weddellii*
Bonaparte Point^19^	S 64.751°, W 64.057°	0.135	9, 17, 25, 29, 36, 43, 50, 55, 57, 62, 67, 73, 80	*M. leonina* , *A. gazella* , *L. weddellii*
Dietrich Island^14^	S 64.739°, W 64.052°	0.076	32, 40, 45, 51, 56, 57, 62, 69, 74, 80	*M. leonina*
Dream Island^2^	S 64.700°, W 64.224°	0.224	44, 68, 81	*M. leonina* , *A. gazella* , *L. carcinophaga* , *L. weddellii*
Elephant Rocks^13^	S 64.743°, W 64.073°	0.034	14, 20, 30, 35, 43, 50, 53, 58, 62, 69, 77, 81, 83	*M. leonina* , *A. gazella* , *L. weddellii*
Humble Island^9^	S 64.739°, W 64.086°	0.094	15, 20, 30, 35, 44, 53, 57, 63, 70, 77, 81	*M. leonina* , *A. gazella*
Kristie Cove^21^	S 64.752°, W 64.044°	0.173	12, 17, 25, 29, 36, 43, 50, 55, 57, 62, 67, 73, 80	*M. leonina* , *A. gazella* , *L. carcinophaga* , *L. weddellii*
Point 8^29^	S 64.752°, W 64.007°	0.122	30, 45, 55, 70	*M. leonina* , *A. gazella*
Point 8 Island^22^	S 64.748°, W 64.004°	0.021	30, 45, 55, 70	—
Shortcut Island^24^	S 64.757°, W 64.042°	0.122	45, 55, 73	*M. leonina* , *A. gazella*
Shortcut Terminus^23^	S 64.754°, W 64.032°	0.035	45, 55, 73	*M. leonina* , *A. gazella*
Torgersen Island^16^	S 64.747°, W 64.073°	0.09	14, 22, 43, 46, 53, 59, 62, 69, 77, 81, 83	*M. leonina* , *A. gazella* , *L. carcinophaga* , *L. weddellii*

*Note:* Habitat suitability modeling used locations of Antarctic fur seals identified on survey dates near peaks in regional abundance across all sites (shaded gray). Site names are accompanied by superscripts that denote site labels used in Figure 1. “Pinniped species identified” summarizes species that we observed across all drone surveys for that site; it does not represent an exhaustive list of known occurrences. Additional ice‐free coastal habitats modeled but not surveyed: Casey Islands^1^, North Wylie Bay^3^, Trivelpiece Island^4^, Fraser Island & shoals^5^, Halfway Island^6^, Breaker Island^7^, Peoples Rocks^8^, Lipps Island^10^, Amsler Island West^11^, Litchfield Island^15^, DeLaca Island^17^, Janus Island & Split Rock^18^, Gamage Point^20^, Spume Island & shoals^25^, Shortcut–Point 8 coastline^27^, Point 8 Adjacency^26^, Eichorst Island^28^, Outcast Islands^30^, Stepping Stones^31^, Christine Island^32^, Dead Seal Island & shoals^33^, Limitrophe Island^34^, islands beyond Dead Seal^35^, Hermit Island^36^, Hellerman Rocks^37^, Cormorant Island^38^, Jacobs Island^39^, Laggard Island^40^, and islands beyond Cormorant^41^. Names in this list might not reflect the current or official place‐names.

### 2.6 Temporal Abundance Modeling

We applied generalized additive modeling with the “mgcv” R package to estimate pinniped abundance as a function of date with and without additional terms, using the Akaike information criterion (AIC) to compare the relative efficacy of models in predicting abundance. Overdispersion was present in all models of count using a Poisson distribution, which we addressed by instead modeling a negative binomial distribution. We first included only date as a predictor of abundance to estimate the overall trend irrespective of survey site with the following model structure:

(1) $$\log\left(\mu \right)=c+f\left(X\right)$$

where μ is the estimated mean count of a survey given an estimated constant c and the day‐of‐year X modified by a smoothing function f (Table A1). We then included date as a predictor with site as a random effect (*n* = 12) to estimate the overall trend when accounting for site‐specific effects with the following model structure:

(2) $$\log\left(\mu_{i}\right)=c+f\left(X\right)+u_{i}+e_{i}$$

where $\mu_{i}$ is the estimated mean count of a survey at site i given an estimated constant c and the day‐of‐year X modified by a smoothing function f, with a random intercept $u_{i}$ and variance $e_{i}$ both corresponding to site i (Table A2). Finally, recognizing that this model with random effects (Equation 2) could not be predicted to sites that had not been surveyed with drones, we modeled abundance as a function of date and suitable area using the following model structure:

(3) $$\log\left(\mu_{i}\right)=c+f\left(X\right)+\log\left(s_{i}\right)$$

where $\mu_{i}$ is the estimated mean count of a survey at site *i* given an estimated constant *c* and the day‐of‐year X, modified by a smoothing function f, with an offset of suitable habitat area $s_{i}$ for site i (Table A3). Suitable habitat area was calculated for sites throughout the study region using satellite products in a MaxEnt model, as described earlier, so this abundance model (Equation 3) was suitable for prediction beyond sites that had been surveyed with drones.

We estimated the date of highest abundance of Antarctic fur seals from the model of abundance as a function of date (Equation 1). We then used the site‐specific model (Equation 2) to estimate abundance at each surveyed site on the date of greatest abundance with a 95% confidence interval (CI) as a function of date and site‐specific effects. We then used the generalizable model (Equation 3) to estimate the abundance at each site with a 95% CI as a function of date and suitable area. We summed the estimated abundance on the date of highest abundance at survey sites, using the second model (Equation 2), with the estimated abundance at the same date for sites not surveyed by drones, using the third model (Equation 3), to produce a region‐wide estimate of terrestrial abundance of Antarctic fur seals during the date of peak terrestrial occupancy in summer 2020.

## 3 Results

### 3.1 Fur Seal Counts and Occupancy Trends

**FIGURE 3:**
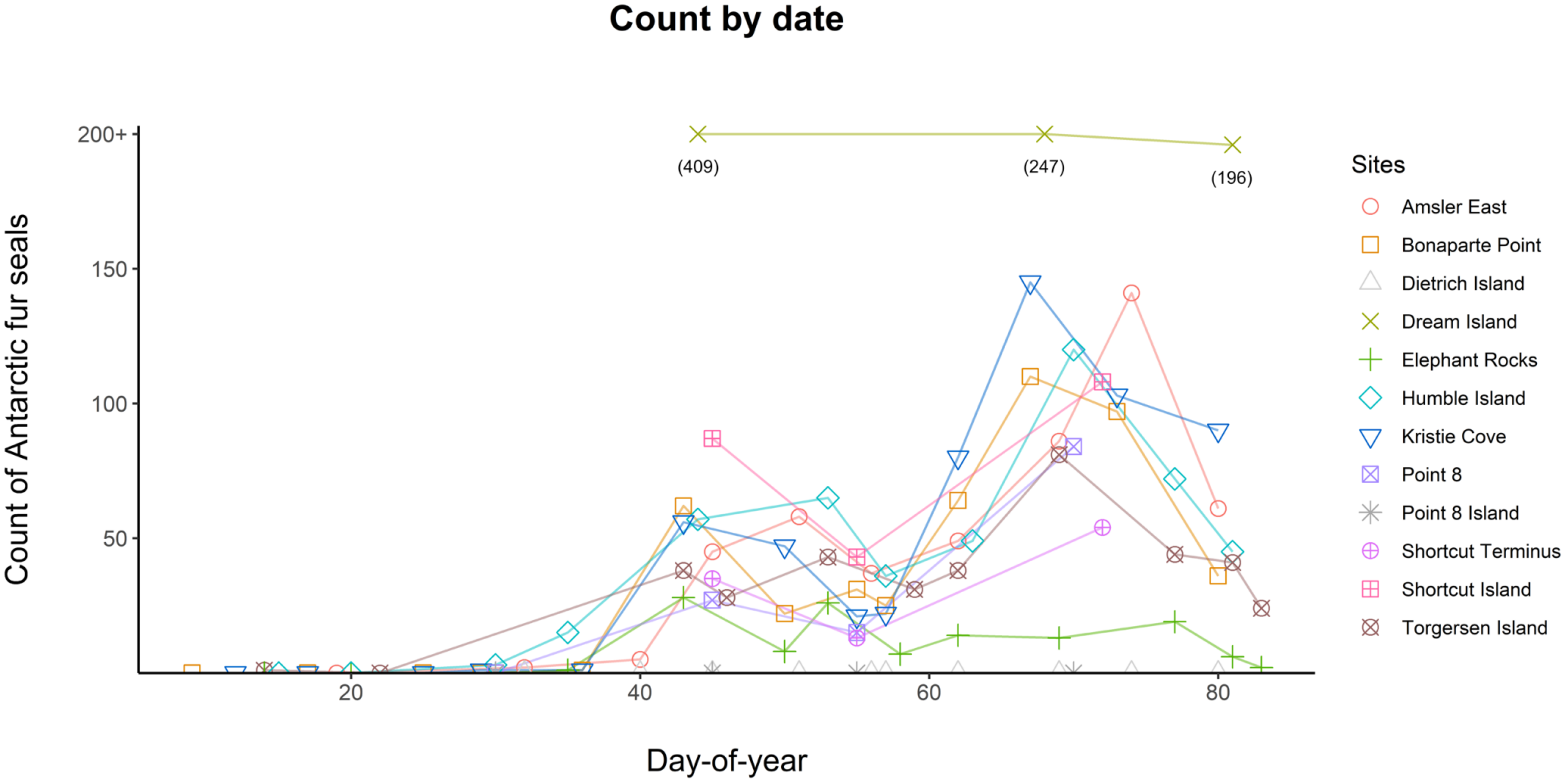
Counts of Antarctic fur seals from drone surveys in the south Anvers Island region during January 9–March 23, 2020. Colored lines link surveys of the same site, but do not necessarily represent the true abundance trend between those surveys.

Antarctic fur seals were readily visible in drone imagery, and resulting counts described a summer arrival of nonbreeding Antarctic fur seals at the end of January and fluctuating abundance throughout the season with shared trends across most sites (Figure 3). Elephant seals, crabeater seals, and Weddell seals were identified at survey sites (Table 1), but not counted or analyzed for this study. Leopard seals were observed swimming at sea or resting on bergy‐bits and the Palmer Station floating dock, and once on a rock offshore of Point 8 during a non‐survey visit.

Fur seals were generally absent from the Palmer Station vicinity until day 29 (January 29), excepting a single fur seal observed at Torgersen Island on day 14 (January 14). The highest count of the season consisted of 409 Antarctic fur seals at Dream Island on day 44 (February 13), while 7 other sites had local highest counts from surveys in the range of days 67–74 (March 7–14, Figure 3). Most sites monitored after this latter peak showed monotonic declines until surveys ceased on day 83 (March 23).

### 3.2 Spatial Characteristics of Fur Seal Habitat Suitability

**FIGURE A4:**
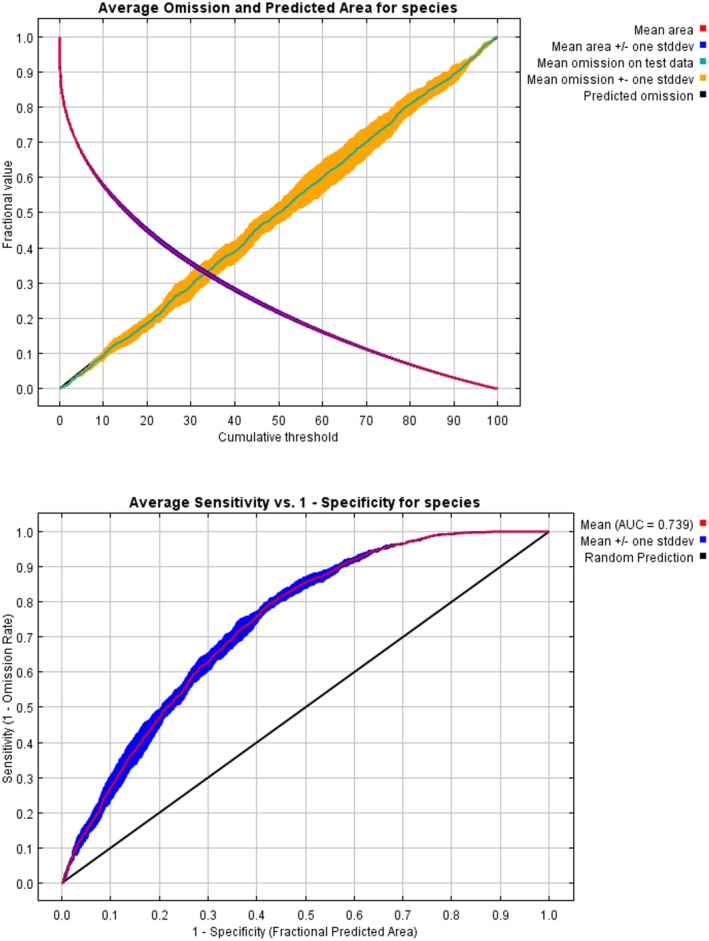
Model performance and sensitivity across cumulative thresholds for the MaxEnt model of habitat suitability. Omission and predicted area (top) are calculated based on training presence records only, as is the receiver operating characteristic (ROC) curve. Means and variance were calculated from 4‐fold (75:25) cross‐validation.

**FIGURE A5:**
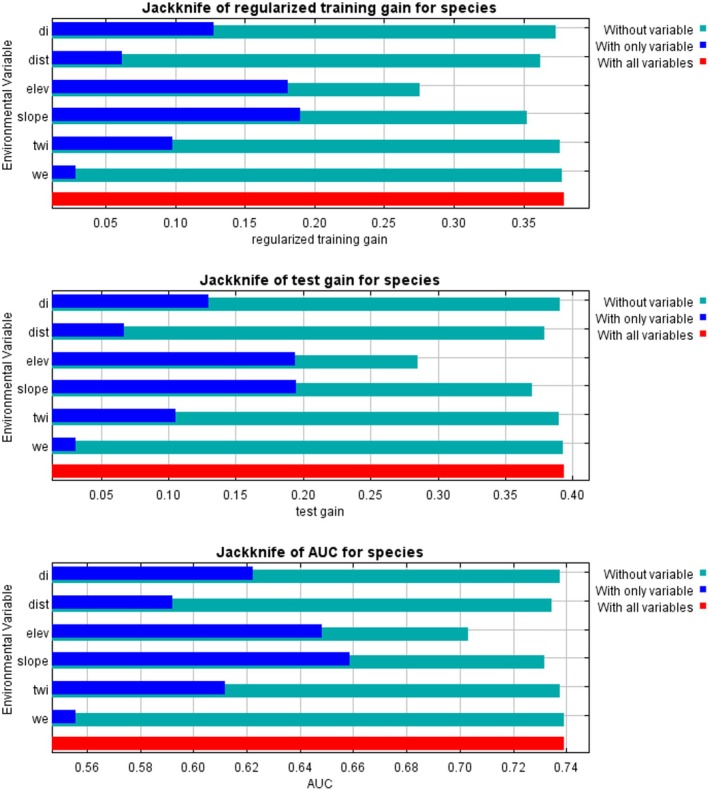
Results of jackknife procedure to estimate predictor importance in the MaxEnt model of habitat suitability. Bars in each panel show the performance of 13 models: the full model (red), models excluding a single predictor (teal) and models using only that predictor (blue). Predictors included potential direct insolation (di), distance to shore (dist), elevation (elev), slope, topographic wetness index (twi) and wind exposure index.

**FIGURE A6:**
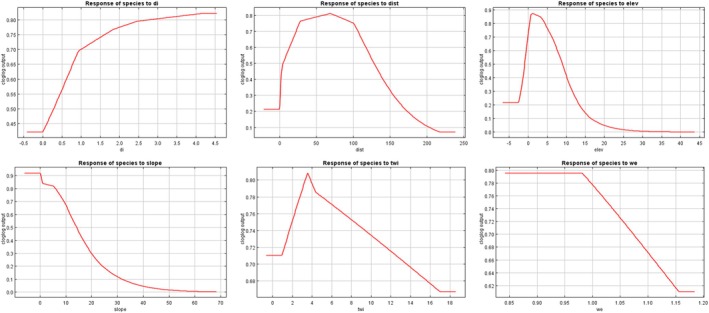
Marginal response curves describing relationships between terrain attributes and habitat suitability, as estimated in the MaxEnt model of habitat suitability, based on a subset of surveyed Antarctic fur seal locations near Palmer Station. Terrain attributes included potential direct insolation (di), distance from shoreline (dist), elevation (elev), slope, topographic wetness index (twi), and wind exposure index (we).

Our MaxEnt model yielded predictions of habitat suitability for the entire region encompassing the Palmer Station vicinity and Wylie Bay (Figure 2) with an AUC of 0.735, indicating moderate separability between suitable and unsuitable habitats (Figure A4). Cross‐validation models yielded a mean AUC of 0.739, indicating consistent performance across data subsets. The predictors that contributed most to the models were elevation (43.5%) and slope (40.8%), followed by distance from shoreline (12%), potential direct insolation (1.4%), topographic wetness (1.4%), and wind exposure (0.8%). These findings were corroborated by jackknife tests of predictor importance (Figure A5), which indicated that slope contained the most useful information (highest gain when used in isolation) and elevation contained the most information not present in other predictors (greatest decrease in gain when omitted); other predictors had variable gains when used in isolation and very minor decreases in gain when omitted. These findings were consistent across training gain, test gain, and AUC metrics. Modeled relationships indicated that seals were most likely to occur in low‐slope (< 15°) and low‐elevation (< 10 m) inshore (25–100 m) terrain, at relatively dry, sun‐exposed, and wind‐sheltered locations (Figure A6). Marginal responses approached 0 at elevations above 30 m, slopes above 60°, and inshore distance of ~210 m, indicating limits of fur seal preference or ability to access such habitats. The threshold of maxSSS (0.5303) yielded a TSS of 0.409, indicating moderate agreement between our classification of “suitable habitat” and our training dataset of fur seal occurrences and pseudoabsences.

### 3.3 Temporal Characteristics of Fur Seal Abundance

**FIGURE 4:**
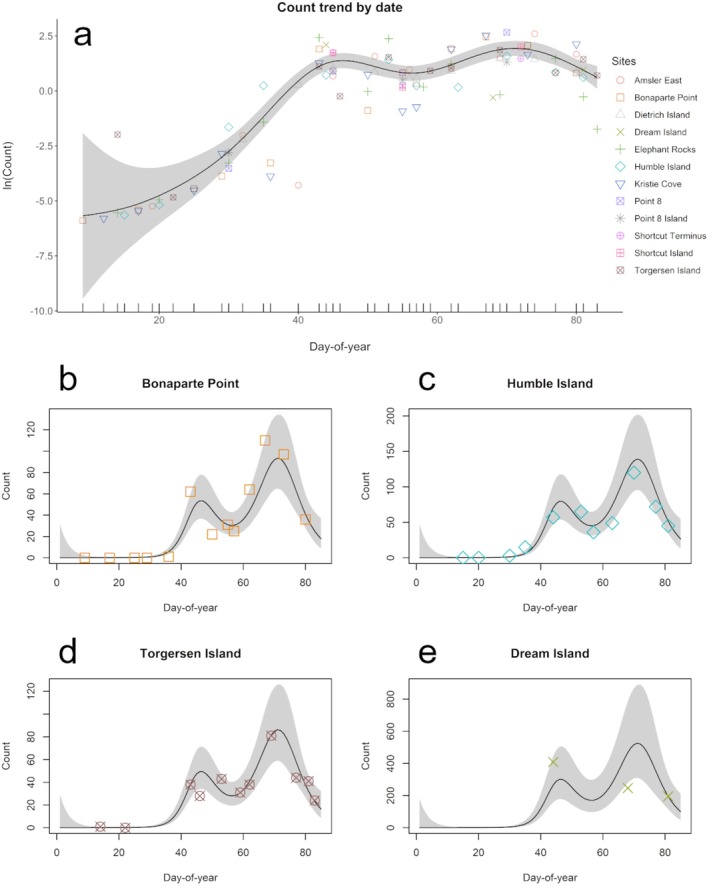
Modeled abundance as a function of date with survey site as a random effect. Model estimates (black lines) with 95% CIs (gray ribbons) were calculated by generalized additive modeling (Equation 2). Model estimates with residuals (a), illustrate the phenology of regional Antarctic fur seal abundance in 2020, while exponentiated predictions with site‐specific random effects (b–e) estimate counts of example sites alongside actual counts for those dates (colored symbols). Estimates of alternative models (Equations 1 and 3) are included in the appendices (Figure A7).

**FIGURE A7:**
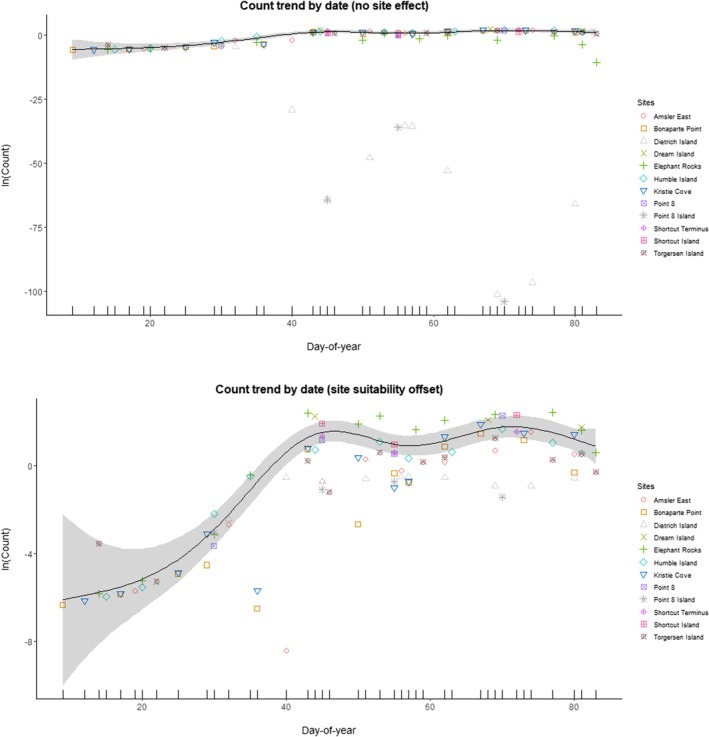
Estimated counts based on abundance models. Generalized additive models were fit to estimate (top) on‐land fur seal abundance in response to only date (Equation 1), and to estimate (bottom) on‐land fur seal abundance in response to date with site suitable area as an offset variable (Equation 3).

Generalized additive models yielded estimates of abundance as a function of date, with and without additional predictors. The simple model of smoothed count in response to date (Equation 1, Figure A7) yielded a fit to our survey counts (AIC = 765, 50.2% deviance explained) that functionally predicted a single distribution of estimates for any site surveyed on a given date in our survey period, regardless of the site's size or suitability. This model nevertheless described the emergent trend in abundance across sites, indicating that two peak abundances of Antarctic fur seals occurred on days 46 (February 15) and 71 (March 11). These peak dates were confirmed in the model that included “site” as a random effect (Equation 2, Figure 4), which yielded a very close fit to our survey counts (AIC 606, 93.7% deviance explained), and in the model that included “suitable area” as an offset variable (Equation 3, Figure A7). The model with suitable area fit our survey counts better than the first model (Equation 1), but not as well as the second model (Equation 2), yielding less confident predictions (AIC 703, 65% deviance explained). Modeling with random effects (Equation 2) estimated a total of 1339 (849–2130, 95% CI) fur seals among drone‐surveyed sites on day 71, whereas modeling with a “suitable area” offset estimated a total of 1083 (703–1669, 95% CI) fur seals for the same date and sites. These estimates closely align with true counts totaling 993 fur seals at surveyed sites between days 68 and 73 (Table 2), straddling the date of peak abundance according to our models. By combining model estimates of surveyed sites using the second model (Equation 2) and sites not surveyed using the third model (Equation 3), we obtained a composite estimated mean of 3560 (2289–5544, 95% CI) Antarctic fur seals on land in the Palmer Station vicinity during peak abundance, day‐of‐year 71 (March 11), in our 2020 study season (Table 2).

**TABLE 2:** Estimates of Antarctic fur seal abundance on the date of peak terrestrial occupancy, day‐of‐year 71 (March 11), during the 2020 study period.

Location	Suitable area (m^2^)	Site suitable area model		Site random effect model		Closest drone survey	
		Count est.	95% CI	Count est.	95% CI	Count	Date [offset from day 71]
Dream Island	64,144	173	112–267	526	311–889	247	68 [−3]
Humble Island	47,956	129	84–199	139	96–202	120	70 [−1]
Shortcut Island	47,256	128	83–196	130	77–220	108	72 [+1]
Kristie Cove	47,360	128	83–197	118	83–169	103	73 [+2]
Amsler Island East	63,448	171	111–264	106	74–152	86	69 [−2]
Bonaparte Point	54,740	148	96–228	93	65–134	97	73 [+2]
Torgersen Island	44,100	119	77–183	86	59–126	81	69 [−2]
Point 8	16,996	46	30–71	59	34–100	84	70 [−1]
Shortcut Terminus	10,712	29	19–45	53	30–91	54	72 [+1]
Elephant Rocks	2396	6	4–10	29	20–43	13	69 [−2]
Dietrich Island	1172	3	2–5	0	0–1	0	69 [−2]
Point 8 Island	972	3	2–4	0	0–3	0	70 [−1]
Summed estimates of surveyed sites, day 71	401,264	1083	703–1669	1339	849–2130		
Summed estimates of sites not surveyed, day 71	822,120	2221	1440–3414				

Entire region						Count est.	95% CI
Composite estimate, day 71						3560	2289–5544

*Note:* Estimates were calculated from the model accounting for suitable area (Equation 3) and the model accounting for site as a random effect (Equation 2) if the site was surveyed. Estimates of individual sites not surveyed sites are included in the appendices (Table A4). A composite estimate summed best estimates (highlighted in gray) to estimate the number of Antarctic fur seals on land during the peak date in 2020.

## 4 Discussion

Antarctic fur seals showed a pattern of seasonal abundance that was common across coastal habitats near Palmer Station, with variance attributable to site‐specific effects. Serial drone surveys provided counts and locations of these seals that were sufficient to characterize distribution and abundance throughout a 3‐month period of mid–late summer 2020. The spatial and temporal modeling of this analysis would likely be improved by the inclusion of additional covariates, including many landscape, seascape, and atmospheric features and processes that were not considered in this study, especially those that are temporally dynamic or that occur at spatial scales larger than our study sites. Nevertheless, these model limitations are assumed to be represented in the wide CIs of our model predictions. Critically, the finite regional and seasonal scope of this study qualify its generalization to other years or regions, but sporadic records from other years and adjacent sites appear to corroborate the overall patterns described in this study.

### 4.1 Past Records Near Palmer Station

Past monitoring at Palmer Station is currently described in coarse resolution, but previous counts suggest a much higher population 30 years prior to our study: our model estimates 198–468 fur seals (95% CI) at Litchfield Island during the peak date of 2020 (Table A4) compared to 874 in 1994 (“Antarctic Specially Protected Area No 113 (Litchfield Island, Arthur Harbor, Anvers Island, Palmer Archipelago): Revised Management Plan” 2014). However, these numbers are comparable only if fur seal distribution can be assumed to be relatively consistent across years. We found that seasonal abundance trends were relatively conserved across sites in 2020 (Figure 4), but we cannot infer interannual consistency from our dataset—although anecdotal observations suggest that many haul‐out sites are reoccupied each year (personal observation). If different haul‐outs show similar trends of seasonal abundance each year then regular counts at key index sites might effectively describe changes in relative abundance within and across years, both in past records (Khoyetskyy and Pishniak 2021; Siniff et al. 2008) and in future monitoring efforts.

Based on past counts from the Palmer Station vicinity and the neighboring Argentine Islands (Khoyetskyy and Pishniak 2021), the predicted date of peak abundance (March 11) in the 2020 monitoring period (January 9–March 23) was likely a close estimate of the peak abundance of the species in that year. Monitoring in the Argentine Islands in 2015 described declining counts after starting surveys in April and estimated that peak abundance in 2016 occurred on March 13 (Khoyetskyy and Pishniak 2021). Similar timing was also described in 1983, with a maximum abundance of 50 Antarctic fur seals in the Palmer Station vicinity on March 24 (Heimark and Heimark 1984), and the maximum local count (874 on Litchfield Island) occurred on March 19, 1994 (“Antarctic Specially Protected Area No 113 (Litchfield Island, Arthur Harbor, Anvers Island, Palmer Archipelago): Revised Management Plan” 2014). The date of arrival for nonbreeding Antarctic fur seals also appears relatively consistent across these records: surveys in the Argentine Islands counted the first Antarctic fur seal of 2016 on January 31, and surveys in 1983 noted their first individual on January 23. Our surveys identified the start of the molt season near January 29, after which most sites were continuously occupied by Antarctic fur seals during the monitoring period (Figure 3). These records together suggest a relatively conserved timing of arrival in late‐January and peak abundance between mid‐March and mid‐April for nonbreeding Antarctic fur seals near south Anvers Island. This constitutes a later seasonal occupancy compared to arrivals and peaks of nonbreeding fur seals in subantarctic islands (Carlini et al. 2006). Large males are occasionally sighted near Palmer Station before January (unpublished data), possibly corroborating our single pupping record (Figure A1) to suggest limited breeding activity in the area during the early summer, but most Antarctic fur seals currently arrive in the later summer during the period associated with the annual molt.

### 4.2 Abundance Drivers

The seasonality of nonbreeding Antarctic fur seals at Palmer Station could reflect both structural and circumstantial drivers of regional terrestrial occupancy. Circumstantial drivers include short‐term weather events, such as wind, snow accumulation and snow melt, climate modes, such as ENSO, and the date of ice retreat or breakout (Waluda, Gregory, and Dunn 2010). Prey availability, which is often influenced by weather and oceanographic conditions, may also consistute a circumstantial driver of Antarctic fur seal occupancy at fine scales (Carlini et al. 2006). Structural drivers include predictable migratory dynamics (e.g., March et al. 2021), given that the most populous and majority of breeding sites of Antarctic fur seals are located far north of Anvers Island, and physiological characteristics, like the timing and energetic demands of the molt, that are unlikely to change quickly between years. Among circumstantial drivers, sea ice retreat usually occurs before the molt period of Antarctic fur seals (Cimino et al. 2023), but nevertheless dense or late sea ice could delay potential breeders in the early spring, and sea ice dynamics shape many subsequent processes in the marine food web (Saba et al. 2014). Short‐term meteorological processes, such as wind and precipitation events, may influence migratory efficiency and on‐land abundance, but multiple years of observations would be necessary to disentangle these interactions.

The seasonal trend that we observed (Figure 4) is likely shaped by the summer migratory process of this nonbreeding fur seal population. Male Antarctic fur seals often disperse from rookeries and nearby haulouts to southerly foraging grounds during the nonbreeding period (Cherel et al. 2009; Kernaléguen et al. 2012; Jones et al. 2020), and the WAP includes key winter habitats for the South Atlantic population (Boyd et al. 1998; Santora 2013; Lowther et al. 2020; March et al. 2021). South Georgia hosts the majority of the world's breeding Antarctic fur seals (Forcada and Staniland 2018), so many or most migratory fur seals on WAP likely originate from those northern rookeries, which is supported by a synchrony of maximum counts that occurred in 1994 at both Palmer Station (“Antarctic Specially Protected Area No 113 (Litchfield Island, Arthur Harbor, Anvers Island, Palmer Archipelago): Revised Management Plan” 2014) and Signy Island (Waluda, Gregory, and Dunn 2010). The timing of arrival at each site is mediated certainly by distance and likely also by seasonal cues, such as temperature and photoperiod, which are thought to trigger other life history events in fur seals (Trites and Antonelis 1994).

### 4.3 Distribution Drivers

Habitat suitability modeling enabled us to estimate where seals might occur in regions not surveyed and on‐land seal abundance during periods of peak terrestrial occupancy. Our findings likely represent preferences or limitations that are particular to Antarctic fur seals; for example, the importance of elevation and slope likely reflects the limitations of otariid terrestrial locomotion, although their association with inland habitats speaks to the species' endurance over navigable terrain. The range of values that seals select and tolerate may also be mediated by the low availability of optimal habitat in this part of Antarctica, forcing compromise in their realized niche. We attempted to model a variety of abiotic environmental conditions using predictors such as wind exposure, potential direct insolation, and topographic wetness index, but notably microclimates in these sites are also influenced by landscape‐scale physical features, such as adjacent mountains, glaciers, underwater canyons, and frontal zones (Convey et al. 2014). Accordingly, this model entails the common provision of spatial modeling: that modeled relationships may perform poorly when applied at farther distances from sites used to train the model. Similarly, habitat selection driven by typical wind exposure or insolation may also change directionally or by intensity across years, further qualifying findings from this single study season. Biotic features, such as moss beds and bird nests, might also influence pinniped site selection positively or negatively at a local scale. These were present at many of the surveyed sites (e.g., Larsen et al. 2024) but were not included in this analysis, and their presence can differ greatly between sites and regions along the WAP. Because this habitat suitability model was trained on locations of nonbreeding, predominantly male Antarctic fur seals, it is likely that breeding females—if they eventually recruit to the region—will select habitat characteristics that deviate from the predictions of our suitability model. But if the seasonal abundance of non‐breeders increases along the deglaciating shorelines of the WAP, our habitat suitability model suggests that they will likely occupy low, flat, inshore terrain, potentially reshaping the local distributions of sympatric flora and fauna in these terrestrial coastal habitats, as has occurred in northern parts of their range (Bonner 1985). Future efforts can improve these habitat suitability estimates by sampling from a broader diversity of locations along the WAP during the peak months of fur seal abundance to capture drivers of occurrence at both local and regional scales.

## 5 Conclusion

Repeated drone surveys successfully yielded spatially explicit counts of Antarctic fur seals in coastal habitats near Palmer Station in 2020. These data, when pooled, revealed distinct preferences among terrestrial habitats and a seasonal abundance trend across sites, with much of the variance between counts explained by site‐specific characteristics. Some variance between counts was successfully explained by each site's area of suitable habitat, and combined distribution and abundance modeling yielded a first estimate of total on‐land abundance of Antarctic fur seals near South Anvers Island during the date of peak abundance in 2020. Even qualified to this region and date, our findings capture fundamental characteristics of the occurrence of Antarctic fur seals during the molt period of late summer. They describe the current state of the species' terrestrial habitat use at the southern edge of its range and establish a baseline from which future studies can evaluate ongoing changes in the range, phenology, and ecological role of Antarctic fur seals in the marine and coastal ecosystems of the WAP. Finally, they demonstrate the efficacy of drone methods for season‐long monitoring of a pinniped population to obtain archivable datasets of digital imagery for replicable counts and fine‐scale distribution and abundance modeling.

## Author Contributions

**Gregory D. Larsen:** conceptualization (lead), data curation (lead), formal analysis (lead), investigation (lead), methodology (lead), project administration (lead), supervision (equal), validation (lead), visualization (lead), writing – original draft (lead), writing – review and editing (lead). **Megan A. Cimino:** formal analysis (supporting), writing – review and editing (supporting). **Julian Dale:** conceptualization (supporting), methodology (supporting), software (supporting), writing – review and editing (supporting). **Ari S. Friedlaender:** conceptualization (supporting), formal analysis (supporting), funding acquisition (equal), investigation (supporting), methodology (supporting), project administration (supporting), resources (equal), supervision (equal), writing – review and editing (supporting). **Marissa A. Goerke:** data curation (supporting), investigation (supporting), resources (supporting), writing – review and editing (supporting). **David W. Johnston:** conceptualization (supporting), data curation (supporting), formal analysis (supporting), funding acquisition (equal), investigation (supporting), methodology (supporting), project administration (supporting), resources (equal), supervision (equal), writing – review and editing (supporting).

## Conflicts of Interest

The authors declare no conflicts of interest.

## Data Availability

Orthomosaic maps from the drone surveys used for this analysis are publicly available in a repository at the following DOI: 10.7924/r4sf2xs2w. Derived products used for analysis, including seal locations, survey metadata, satellite‐based maps, and habitat suitability outputs, are publicly available in a repository at the following DOI: 10.5061/dryad.qv9s4mwp0.

## Appendix 1 Drone Imagery and Collection

Drone flights were conducted using DJI (Da‐Jiang Innovation, Shenzhen, Guangdong, China) Phantom 4 Pro quadcopter drones with the default gimbal‐stabilized camera payload (1″ CMOS sensor, 5472 × 3648 pixels) capturing reflectance in digital red, green and blue bands. Surveys were flown targeting a minimum of 65% overlap at altitudes targeting 1.5‐cm GSD or 75% minimum overlap for 3‐cm GSD (55 and 110 m altitude, respectively) relative to sea level because reliable elevation base maps were not available on hand at the time of survey. Overlap and GSD therefore varied by photograph, depending on the actual elevation below at each photograph site, and rare but occasional gaps occurred in coverage if the camera delayed or lapsed between consecutive photographs. Flight plans were designed and carried out using Universal Ground Control Station (Baloži, Latvia) software version 3.3.438 on a laptop alongside the UGCS for DJI app on a tablet, and drones were launched and recovered by hand from small boats, adjacent landmasses, or local peaks to maintain line‐of‐sight throughout the survey. If sites were directly accessed for surveillance, care was taken to avoid or minimize all possible animal disturbance, and nearby seal locations were noted or photographed at ground level upon landfall, so undisturbed locations could be used instead if they were subsequently disturbed by flight operations or adjacent research or logistical operations that sometimes co‐occurred.

Flight plans consisted of parallel overlapping transects, repeating for either the extent of the target survey region, or as much as could be covered in the duration of a single battery. Transect orientation targeted each region's longest axis to maximize battery efficiency, since drones decelerated toward waypoints and therefore spent more time proportionally on turns than on straight transects. Depending on the target GSD, larger sites often required multiple flights and batteries to survey the entire site with a single grid of overlapping aerial photography. Surveys consisted of at least one grid of aerial photography targeting 1.5‐cm GSD with ~65% overlap, to capture a complete orthomosaic of land cover and pinniped locations in high resolution, and within the campaign each site received at least one survey that also included a second consecutive grid of aerial photography targeting 3‐cm GSD with ~75% overlap, and in most cases additional oblique photography collected around the perimeter of the site, to provide robust coverage for photogrammetric surface modeling.

Images with timestamps and GPS metadata were collected and stored in removable memory aboard the aircraft during each flight; position and system data were also telemetered to the ground control station over radio frequencies where they were actively monitored and logged. Surveys were carried out under amenable flight conditions that entailed winds > 25 mph, clear visibility > 3 miles, and little or no precipitation. All flights took place during daylight hours on an opportunistic schedule, preferentially targeting overcast conditions with diffuse lighting and shadows.

## Appendix 2 GPS Survey and Ground‐Truthing

We selected three sites opportunistically for georeferencing and ground‐truth comparisons—Humble Island, Torgersen Island, and Elephant Rocks—with a survey‐grade Global Navigation Satellite Systems receiver using differential corrections from the adjacent PAL2 base station (Johns 2006). Ground truth surveys used a system of natural GCPs, consisting of semipermanent natural features, such as boulders, peaks, and cracks in bedrock, that could be located precisely in drone imagery and approximately in satellite imagery. GCPs were used to confirm the accuracy of DEMs from satellite products, and we corrected DEMs to ground‐truth elevation values before using these data for habitat modeling.

Ground truthing was conducted using a Trimble R7 system (Sunnyvale, California) with a Zephyr Geodetic Base L1/L2 Antenna (part number 41249‐00). Points were averaged over 10 s with PDOPs of ≤ 2.57, mostly < 2.0, and were post‐processed for differential corrections using records from the nearby PAL2 base station (Johns 2006) for nominal 10 mm + 1 ppm RMS horizontal precision and 20 mm + 1 ppm RMS vertical precision. We identified candidate GCPs from orthomosaics collected early in the season, targeting features that appeared high‐contrast (ideally visible in imagery from both drones and satellites), relatively permanent (not likely to change within or across seasons), and relatively flat (less vulnerable to orthorectification errors). Candidate GCPs were selected by hand to achieve evenly spaced coverage across each study site. Survey sites were chosen opportunistically based on map availability, biological interest, and ease of access. During the GPS survey, we located and accessed as many of these features as we could within logistical constraints, and we surveyed each accessed feature using the GPS system.

## Appendix 3 Orthomosaics, Elevation Models, and Terrain Products

Orthomosaics and DSMs were generated in Pix4D software version 4.3 using a customized “3D Maps” workflow with highest settings, image matching using triangulation of image geolocation and image similarity with a maximum of one pair for each image, and “Geometrically Verified Matching” strategy for matching image pairs. Sites that received ground truth surveys were processed using differentially corrected locations of GCPs. All DSMs were generated without smoothing or noise filtering, and from those models, the orthomosaics were derived without radiometric processing for calibration corrections.

We selected pairs of reference‐quality DSM and orthomosaic base maps from surveys that included multiple grids of aerial photography for maximum coverage. When multiple candidate surveys were available for a site, we visually inspected all DSMs and selected the model with the most complete coverage and fewest visible artifacts, such as interpolated gaps or unnatural disjunctions in the landscape. We then co‐registered orthomosaics from other surveys to the base map orthomosaic using automatically generated control points, which we reviewed for accuracy, before warping with a third‐order polynomial transformation, using ArcGIS Pro 2.7.1. We then marked seal locations on the selected base maps and aligned orthomosaics. We also cropped all orthomosaics for a given site to a mask of shared coverage.

For terrain analysis, we used two satellite DEM products from stereo imagery provided by the DigitalGlobe collection to the Polar Geospatial Center. The scenes consisted of one DEM from WorldView‐3 imagery collected on November 19, 2019, and processed using SETSM version 4.3.7, and one DEM from WorldView‐1 imagery collected on October 20, 2012, and processed using SETSM version 4.3.6, respectively named “WV03_20191119_104001005589A700_1040010054AE7700_2m_lsf_v040307” and “WV01_20121020_102001001D99B900_102001001EB08100_2m_lsf_v040306.”

These scenes were selected based on visual assessment, ensuring that areas of exposed land did not include obvious elevation errors, which were present to various degrees in parts of all 27 DEMs received from Polar Geospatial Center—although many DEMs included large usable regions where land elevation estimates appeared accurate. Most study sites were modeled successfully in both selected products, and we ensured that all sites that included glacial remnants and interfaces were described by the more recent 2019 DEM, if possible. We overlaid DEMs with drone DSMs to ensure that they were closely aligned horizontally, such that seal locations identified in orthomosaics were appropriately registered to satellite DEMs, then we extracted DEM values at each ground‐truthed GCP to estimate offsets of reference DSMs and each satellite DEM. We corrected each DEM by its mean offset, −1.66 for the 2019 DEM and +1.57 for the 2012 DEM.

Ground truth surveys at Humble Island, Torgersen Island, and Elephant Rocks showed high vertical accuracy among drone‐derived DSMs (−0.20 ± 0.49 m), which were generated using the GCPs, and larger offsets in our selected DEMs from WorldView‐3 in 2019 (−1.66 ± 0.73 m) and WorldView‐1 in 2012 (1.57 ± 0.68 m). However, while we used drone‐derived DSMs to visually assess satellite DEM products, we did not use them for habitat analyses, despite their higher accuracy and precision, because they were not available for non‐surveyed parts of our study region and were not necessarily representative of the region‐wide data derived from satellite imagery. Visual comparison between satellite‐derived DEMs and down‐sampled drone‐derived DEMs suggested that large‐scale topographic features (coastlines, hills, valleys) aligned spatially between the two modalities, but fine‐scale texture and contrasts (chasms, peaks, terrain rugosity) were smoothed in satellite‐derived DEMs, likely as a result of their lower source GSD and algorithmic interpolations (Figure A3).

We generated preliminary land‐masks for each surveyed site using DSMs from drone surveys, creating polygons at mean sea level (−1.129 m EGM96), then we generated land‐masks for analysis from corrected satellite imagery by selecting an elevation threshold that yielded similar boundaries for all surveyed sites and new polygons for sites not surveyed. Based on similarity to drone land‐masks, we predominantly used the land‐mask from the corrected 2012 DEM, with an empirically selected threshold of 0 m EGM96 (Figure A3), except at sites with receding coastal glacial features, for which we used the corrected 2019 DEM if available for that site, with a selected threshold of −1.129 m EGM96 (MSL). We visually inspected the combined land‐mask and manually edited it, if errors were visible, and masked it to exclude the Marr Ice Piedmont extent in 2019. We applied the land‐mask to each DEM with a buffer of 10 m to enable neighborhood‐based calculations at the edges of the unbuffered land‐mask, and mosaicked the land regions that were only modeled in the 2012 DEM together with the rest of the land regions that were modeled in the 2019 DEM.

We created terrain products by applying topographic GIS tools to the mosaicked, buffer‐masked DEM, and then again masked the products to the unbuffered land‐mask, to obtain an identical complete extent of the land‐mask for each product. Elevation was obtained directly from the corrected, mosaicked DEM, and slope was calculated using the appropriate ArcGIS tool. Distance from shore was calculated using the “distance accumulation” tool in ArcGIS, including elevation as a surface raster for surface distance, and using a land‐and‐glacier polygon to calculate distance from shore (such that inland glacier interfaces were not treated as shoreline boundaries). Potential direct insolation was calculated using the “potential incoming solar radiation” tool in SAGA 8.2.1 with solar positioning for the date of March 11, 2020. Wind exposure was calculated using the “wind exposition index” tool in SAGA 8.2.1 with 15° increments of wind angle and a 10‐m search radius. Topographic wetness was calculated using the “one‐step topographic wetness index” workflow in SAGA 8.2.1.

**TABLE A1:** Model results of a generalized additive model (Equation 1) estimating abundance as a smoothed function of date.

Intercept Estimate	Standard error	*Z*‐score	*p*
2.587	0.183	14.14	< 0.001

Approximate significance of smooth terms			
Term	Effective degrees of freedom	Chi‐squared statistic	*p*
Date	5.541	93.63	< 0.001

Model summary			
*R*‐squared (adjusted)	Deviance explained	REML score	AIC
0.192	50.2%	384.45	764.7071

*Note:* The model was trained on all surveys of study sites (*n* = 98), and abundance was modeled using a negative binomial distribution with *θ* = 0.59 and a log link function.

**TABLE A2:** Model results of a generalized additive model (Equation 2) estimating abundance as a smoothed function of date, with site as a random effect.

Intercept Estimate	Standard error	*Z*‐score	*p*
1.6582	0.7948	2.086	0.037

Approximate significance of smooth terms			
Term	Effective degrees of freedom	Chi‐squared statistic	*p*
Date	6.713	212.7	< 0.001
Site	10.638	179.9	< 0.001

Model summary			
*R*‐squared (adjusted)	Deviance explained	REML score	AIC
0.688	93.7%	329.49	606.3818

*Note:* The model was trained on all surveys of study sites (*n* = 98), and abundance was modeled using a negative binomial distribution with *θ* = 5.83 and a log link function.

**TABLE A3:** Model results of a generalized additive model (Equation 3) estimating abundance as a smoothed function of date, with suitable area as an offset.

Intercept Estimate	Standard error	*Z*‐score	*p*
−7.7012	0.1598	−48.2	< 0.001

Approximate significance of smooth terms			
Smooth term	Effective degrees of freedom	Chi‐squared statistic	*p*
Date	5.977	128.4	< 0.001

Model summary			
*R*‐squared (adjusted)	Deviance explained	REML score	AIC
0.478	65%	355.04	702.7576

*Note:* The model was trained on all surveys of study sites (*n* = 98), and abundance was modeled using a negative binomial distribution with *θ* = 1.329 and a log link function.

**TABLE A4:** Estimates of peak abundance of Antarctic fur seals on land at individual sites not surveyed by drones.

Site	Suitable area (m^2^)	Count estimate	95% Confidence interval
Litchfield Island^15^	11,2704	304	198–468
Christine Island^32^	78,176	211	137–325
Hermit Island^36^	76,940	208	135–320
Janus Island & Split Rock^18^	62,420	168	109–259
Amsler Island West^11^	60,584	164	106–252
Limitrophe Island^34^	44,132	119	77–183
Casey Islands^1^	40,300	109	71–167
Outcast Islands^30^	40,272	109	71–167
Fraser Island & shoals^5^	39,464	107	69–164
Gamage Point^20^	35,228	95	62–146
Laggard Island^40^	29,864	81	52–124
Peoples Rocks^8^	26,448	71	46–110
Dead Seal Island & shoals^33^	25,540	69	45–106
Halfway Island^6^	24,240	65	42–101
Cormorant Island^38^	18,412	50	32–77
Breaker Island^7^	12,488	34	22–52
Hellerman Rocks^37^	12,220	33	21–51
North Wylie Bay^3^	11,840	32	21–49
Lipps Island^10^	10,452	28	18–43
Jacobs Island^39^	10,124	27	18–42
Stepping Stones^31^	8772	24	15–36
Spume Island & shoals^25^	8616	23	15–36
Eichorst Island^28^	7712	21	14–32
DeLaca Island^17^	6124	17	11–25
Islands beyond Cormorant^41^	4844	13	8–20
Trivelpiece Island^4^	4360	12	8–18
Point 8 Adjacency^26^	4360	12	8–18
Islands beyond Dead Seal^35^	2956	8	5–12
Shortcut–Point 8 coastline^27^	2528	7	4–11

*Note:* Estimates were predicted using the MaxEnt model for suitable topography to estimate ‘suitable area’ with a generalized additive model (Equation 3) for day‐of‐year 71. Site subscripts denote labeled locations in Figure 1.
